# Retraction Note: C-X-C chemokine receptor family genes in osteosarcoma: expression profiles, regulatory networks, and functional impact on tumor progression

**DOI:** 10.1186/s41065-026-00752-0

**Published:** 2026-10-17

**Authors:** Siqi Dong, Han Xu, Xianglei Kong, Yanchang Bai, Xijun Hou, Fei Liu, Yan Xu

**Affiliations:** 1 Orthopedics Department, First Hospital of QinHuangdao, QinHuangdao, 066000 China; 2 Department of Oncology, First Hospital of QinHuangdao, QinHuangdao, 066000 China; 3 Department of Neurology, First Hospital of QinHuangdao, QinHuangdao, 066000 China; 4 Hospital Infection Department, First Hospital of QinHuangdao, QinHuangdao, 066000 China

**Retraction Note: Hereditas 162, 194 (2025)**

**https://doi.org/10.1186/s41065-025-00569-3**

The Editor-in-Chief has retracted this article. After publication, concerns were raised regarding the in vitro images presented in Figs. 8, 9 and 10 of this article, where the patterns of cells and colonies do not appear consistent with natural cell growth and proliferation. Additionally, the uncut western blot gel presented in the Supplementary Material appears to show contradictory results, with two proteins probed in adjacent lanes with different results. It has also been noted that a number of references in this article do not appear to support the statements preceding the citations.

The Editor-in-Chief therefore no longer has confidence in the presented data.

Yan Xu and Siqi Dong have not explicitly stated whether they agree or disagree with the retraction. The other authors have not responded to correspondence regarding this retraction.

